# Sodium Bicarbonate In In-Hospital and Out-of-Hospital Cardiac Arrest: A Systematic Literature Review

**DOI:** 10.7759/cureus.68192

**Published:** 2024-08-30

**Authors:** Pedro Manuel Batarda Sena, Joao Rodrigues, Francisco Das Neves Coelho, Brenda Soares Nunes, Orlando Fernandes, Nicodemos Fernandes, José J Nóbrega

**Affiliations:** 1 Intensive Care Department, Hospital Central do Funchal, Funchal, PRT; 2 Helicopter Emergency Medical Service, Instituto Nacional de Emergência Médica, Lisbon, PRT; 3 Medical Emergency and Resuscitation Vehicle of the Local Health Unit of Arco Ribeirinho (VMER ULSAR), Instituto Nacional de Emergência Médica, Barreiro, PRT; 4 Pulmonology Department, Centro Hospitalar de Lisboa Central, Lisbon, PRT; 5 Helicopter Emergency Medical Service, Instituto Nacional de Emergencia Medica, Lisbon, PRT; 6 Intensive Care Department, Hospital Egas Moniz, Centro Hospitalar de Lisboa Ocidental, Lisbon, PRT

**Keywords:** cardiopulmonary resuscitation (cpr), in-hospital cardiac arrest, cardiac arrest, out-of-hospital cardiac arrest, sodium bicarbonate

## Abstract

Cardiac arrest is a common cause of death worldwide. Sodium bicarbonate (SB) has commonly been used during cardiopulmonary resuscitation (CPR) to correct metabolic acidosis (MA). However, the existence of evidence about its administration remains controversial. This systematic review aimed to summarize the effectiveness of SB in patients with in-hospital and out-of-hospital cardiac arrest. We searched Medline, Scopus, and the Cochrane Central Register of Controlled Trials (CENTRAL) for studies that used SB in cardiac arrest, from November 1962 until December 2023. A total of 372 records were identified and 12 studies were included. Despite few studies suggesting that SB may improve outcomes in prolonged CPR, the overall data revealed that SB was associated with lower rates of ROSC and outcomes. This review conceded that there is limited evidence to warrant the use of SB during CPR other than under specific conditions, which include hyperkalemic cardiac arrest, severe cardiotoxicity, or overdose due to tricyclic antidepressants. In conclusion, SB is not recommended for conventional use in patients with cardiac arrest. Further studies should be performed to determine whether it has any benefit in these scenarios.

## Introduction and background

Sodium bicarbonate (SB) has frequently been used in the treatment of severe metabolic acidosis (MA) in patients undergoing cardiac arrest [1]. MA refers to the buildup of acid in the body in the event of kidney failure or disease, resulting in acid-base imbalance [2]. To ensure a meaningful end to resuscitation, the intracellular and extracellular pH has to be normalized. As a result, the correction of MA using SB became the practice as was suggested by the Advanced Cardiac Life Support (ACLS) publication guidelines of 1976, making SB the most commonly utilized medication following incidents of cardiac arrest up to the middle of the 1980s [3].

The belief that using SB during cardiac arrest-corrected MA was based on studies conducted in dogs in 1973, 1982, and 1994 [1]. In the 1973 study in which dogs received low, average, and high concentrations of SB, the researchers found that the severity of MA determined the use of SB as a requirement for resuscitation and that the underlying SB concentration in the plasma membrane influenced the apparent distribution of SB [4]. Similarly, in the 1982 study, which involved dogs with lactic acidemia induced by phenformin, the researchers concluded that SB reduced blood flow into the portal vein, pH of the liver, cardiac output, and erythrocytes [5]. Finally, in the 1994 study, which investigated the efficiency of SB versus Carbicarb versus hypertonic saline in hemorrhagic shock in dogs, the researchers found that cardiac index, blood pressure, oxygen consumption, and oxygen delivery improved in all groups, without significant improvement in the SB and Carbicarb-treated animals, despite their excellent acid-base status [6].

Nevertheless, increasing concerns over its potential benefits versus harmful effects resulted in a decline in its use to almost no use by 1991 [1]. Currently, the application of SB in patients experiencing cardiac arrest has raised a heated debate and controversies, making its frequency of use vary significantly from one medical center to another, following the 2010 publication by the American Heart Association (AHA) prohibiting the application of SB for individuals in cardiac arrest. However, the AHA proposed that SB may be used as necessary for hemodynamic stability when administered in boluses of 1 mL/kg and QRS narrowing in incidents of hyperkalemic cardiac arrest, acute cardiotoxicity (heart damage), or overdose due to tricyclic antidepressants [7].

In the US, over 200,000 individuals undergo in-hospital cardiac arrest (IHCA) annually, making CPR commonplace in such settings [8]. Other authors approximated this to 290,000 individuals annually, furthering that the IHCA mortality rate ranges between 9% and 13%, with a one-year survival [9]. However, in Europe, the annual prevalence of IHCA is about 150 to 280 per 100,000 hospital admissions, with survival to hospital discharge averaging 25% (15-34%) [10]. On the other hand, the overall rate of survival for out-of-hospital cardiac arrest (OHCA) in the US is 26.3% to hospital admission and 9.6% to hospital discharge [11]. In Europe, the OHCA annual prevalence stands at 67 to 170 per 100,000 individuals, with survival to hospital discharge averaging 9% (0-18%) [10].

In addition, there are limited types of medications for use during cardiac arrest, and their frequency of use during resuscitation has become debatable due to the little empirical support that reinforces their capacity to improve outcomes [8,11]. Some of the medications used during resuscitation include epinephrine, calcium chloride, and SB, which have shown little to no benefit in IHCA and OHCA patients with reference to survival to hospital admission and survival to hospital discharge [8]. In 2015, the largest study up to then was conducted for the first time on patients with IHCA as initial studies were conducted on patients without cardiac arrest to test the efficacy of SB and calcium carbonate (CC) in hyperkalemic cardiac arrest [12]. The researchers established that the prevalence rate of hyperkalemia was 12-21.4% in IHCA and that SB increased return of spontaneous circulation (ROSC), but the data did not translate to the low survival rates observed in 9% of the patients at 24 hours [13].

This systematic literature review aims to evaluate the evidence surrounding the efficacy of SB during CPR procedures in patients undergoing both IHCA and OHCA and to clarify the conditions under which SB administration is justified.

Methods

**Figure 1:**
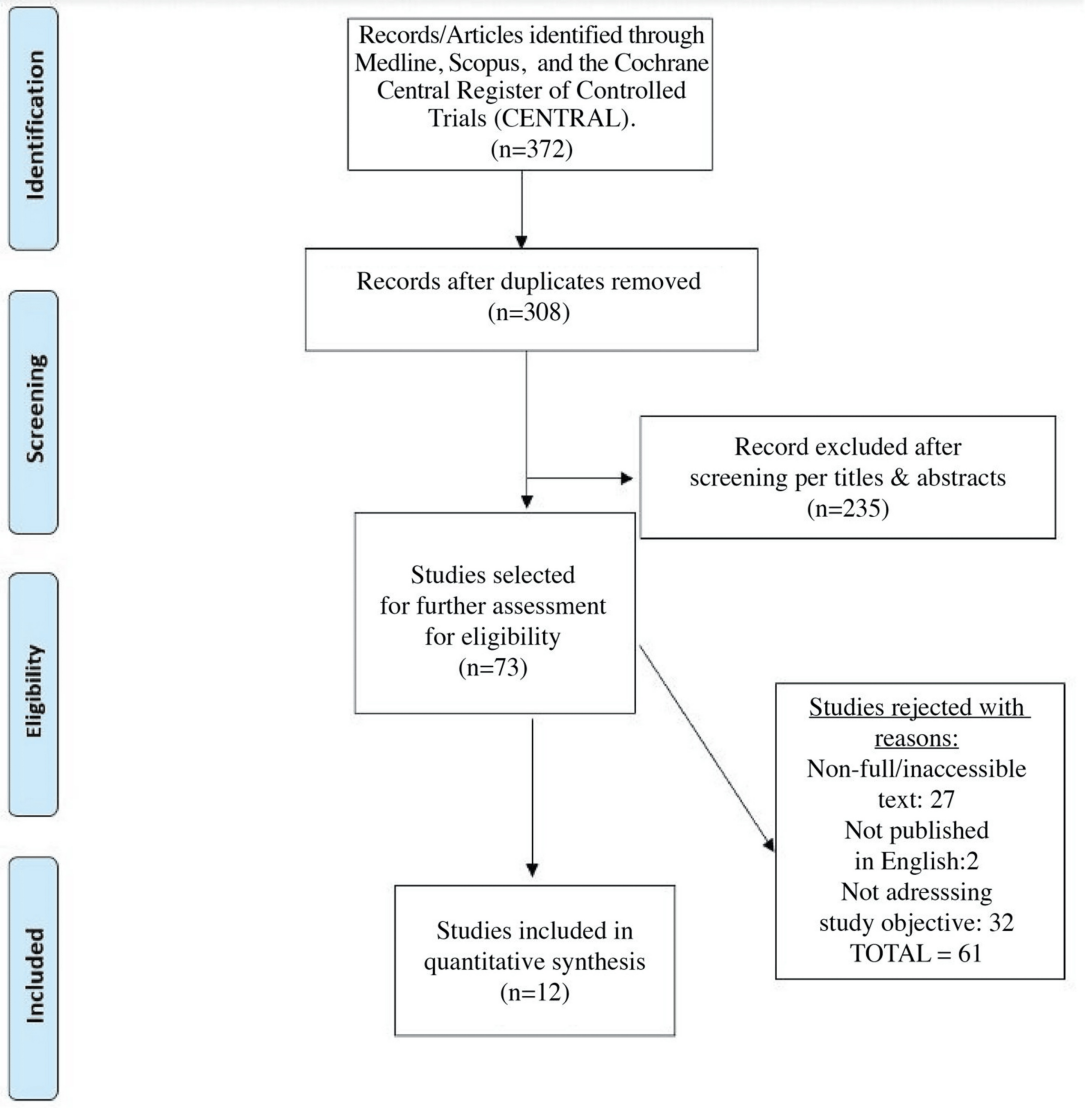
Preferred Reporting Items for Systematic Reviews and Meta-Analyses (PRISMA) flow diagram of the selection process.

The present study was conducted according to the Preferred Reporting Items for Systematic Reviews and Meta-Analyses (PRISMA) guidelines, with the protocol and checklist summarized in Figure 1 [14].

Because the study was based on and informed by insights and conclusions gleaned from extant secondary sources of information, no institutional or formal ethical approval was needed.

Study identification and eligibility criteria

Our systematic review meticulously searched Medline, Scopus, and the Cochrane Central Register of Controlled Trials (CENTRAL) for studies on the use of SB in cardiac arrest, from the inception of the databases in November 1962 to December 2023. The search utilized keywords such as "out-of-hospital cardiac arrest," "heart arrest," "in-hospital cardiac arrest," "cardiac resuscitation," "cardiopulmonary resuscitation," "sodium bicarbonate," and "bicarbonate" to ensure a broad capture of relevant literature. Further depth was added to our search by manually reviewing the bibliographies of included trials and related review articles for additional pertinent references. This review considered all studies described as retrospective or prospective observational studies and randomized controlled trials, published in English, with abstracts and full-text articles available.

Study selection

We included studies that were randomized controlled trials (RCTs) or observational studies, examined the use of SB in adult patients (≥18 years) experiencing cardiac arrest, and reported on outcomes such as ROSC, survival to hospital admission, survival to discharge, and neurological outcomes. We also included review articles with the main purpose of deepening the understanding of the specific role of SB in cardiac arrest. These reviews took place many years ago and the majority of studies taken into account were substantially different from those included in our review. Reviews analyze and synthesize a lot of evidence, making it beneficial when original sources are not available or accessible. That said, we considered that the aggregated results can mainly be used for contextualization and additional references. 

We excluded case reports, case series, and animal studies; studies focusing on specific subpopulations (e.g., pre-existing acidosis, intoxication, or drug abuse); and studies not published in English or without accessible full texts. An essential requirement for inclusion was that the studies must compare outcomes between treatment arms, one with SB administration and the other without.

Study selection and extraction

Our search strategy included terms related to cardiac arrest and sodium bicarbonate. Keywords used were "out-of-hospital cardiac arrest," "heart arrest," "in-hospital cardiac arrest," "cardiac resuscitation," "cardiopulmonary resuscitation," "sodium bicarbonate," and "bicarbonate."

The information extracted was multifaceted and included the study’s bibliographic information, type of study, objectives, and findings. Details of the literature search and data screening were based on the PRISMA flow diagram (Figure 1). A descriptive approach was used to examine the data extracted, and the studies were analyzed based on the outcomes assessed and their relevance to the study’s objective.

The graph shows the PRISMA flow diagram [15] reporting the literature search process and the reasons for article inclusion and exclusion. Overall, the search and selection process was done in two main steps: (1) title and abstract screening using the inclusion criteria set and (2) relevance review of full-text articles from the first phase.

## Review

Results

Altogether, a total of 372 records were identified. After removing 64 duplicate records, 308 studies were assessed for inclusion and screened by title and abstract for relevance. As a result, 235 records were excluded, and 73 articles were consequently screened in full text. Lastly, 12 studies were included in the presented SLR (Table 1). This was after removing 61 articles for not meeting the eligibility criteria - 27 were non-full and inaccessible, two were non-English, and 32 did not address the study’s objective [15]. 

**Table 1:** Selected studies and reviews on the use of SB during cardiac arrest before 2023. MA, metabolic acidosis; SB, sodium bicarbonate; ACLS, Advanced Cardiac Life Support; CPR, cardiopulmonary resuscitation; ROSC, return of spontaneous circulation; TP, trometamol-phosphate; OHCA, out-of hospital cardiac arrest; ALS, Advanced Life Support

Year	Authors	Type of study	Aim	Findings	Conclusion
1990	Roberts et al.	Retrospective study	Mortality predictors during cardiac arrest	The low survival rate in patients who received SB	The low survival rate was attributed to severe MA in patients who received SB.
1995	Stiell et al.	Observational cohort study	The correlation between ACLS drugs (SB) and 1-hour survival rate to hospital discharge	The correlation between SB and survival is not significant but the timing of administering the drug was a critical factor.	SB is effective in prolonged CPR to treat MA and improve ROSC.
1995	Dybvik et al.	RCT	The difference between 0.9% saline solution and SB mixed with trometamol-phosphate (TP) in OHCA patients	Buffer therapy had no significant improvement in outcomes in MA patients.	SB does not show significantly improved outcomes for MA patients even when mixed with TP.
1998	Walraven et al.	RCT	The correlation between ACLS drugs (SB) and survival rates in hospital discharge	There was no correlation between SB and successful resuscitation.	There is insufficient proof to support the application of SB in resuscitation.
1999	Adgey & Johnston	Review	Conditions for use of SB during cardiac arrest	There is a limit to which buffer solutions should be used during resuscitation	SB should be administered after prolonged CPR or in hyperkalemia or tricyclic antidepressant overdose.
1999	Datta et al.	Review	Assess the use of SB in ALS, life support, and post-resuscitation care	SB ought not to be used customarily for cardiac arrest patients.	SB is not suggested for regular use in cardiac arrest patients.
2002	Bar-Joseph et al.	Retrospective study	Determine the correlation between SB and ACLS duration	A linear correlation exists between SB and ACLS.	SB should be initiated early due to the development of severe MA.
2005	Bar-Joseph et al.	Retrospective study	Determine the correlation between SB and ACLS duration	The duration from the onset of cardiac arrest to the initiation of ACLS was less than 30 minutes.	Improved ROSC and better outcomes are associated with early and frequent administration of SB.
2006	Vukmir & Katz	RCT	The difference in survival between patients who received SB after ACLS protocol and those who received normal saline	No difference in survival between patients who received SB after ACLS protocol and those who received normal saline.	SB does not show significantly improved outcomes in cardiac arrest patients.
2008	Spohr et al.	Review	The ACLS medications with beneficial short-term survival after cardiac arrest	SB should not be used routinely for cardiac arrest patients.	SB should only be recommended in situations where lengthy resuscitations have failed.
2013	Weng et al.	Retrospective cohort study	The difference in survival between patients who received SB after ACLS protocol and those who received normal saline	A higher ROSC percentage was noticed in patients who received SB in prolonged CPR.	Patients who received SB in protracted CPR have a higher percentage of ROSC than patients who received normal saline.
2018	Touron et al.	Retrospective cohort study	To obtain information of assistance in designing a randomized controlled trial of bicarbonate therapy after OHCA in specific patient subgroups	Sodium bicarbonate was associated with a lower likelihood of favorable functional outcomes at hospital discharge.	Prehospital sodium bicarbonate administration was not associated with neurolo+A1:F14gic outcomes in a French dataset and was associated with worse neurologic outcomes in a North American dataset.

To ensure the robustness and transparency of our systematic review, we conducted a risk of bias assessment for all included studies. This assessment utilized the Cochrane Risk of Bias Tool for RCTs and the Newcastle-Ottawa Scale for observational studies. Below is a detailed report of the risk of bias assessment for the included studies that we resume in Table 2.

**Table 2:** Risk-of-bias assessment.

Study	Study Type	Selection Bias	Performance Bias	Detection Bias	Attrition Bias	Reporting Bias	Overall Bias
Roberts et al. 1990	Observational	High	Unclear	High	Low	High	High
Stiell et al. 1995	Observational	Low	Low	Low	Low	Low	Low
Dybvik et al. 1995	RCT	Unclear	Low	Low	Low	Low	Low
Walraven et al. 1998	RCT	Low	Low	Low	Low	Low	Low
Adgey & Johnston 1999	Review	N/A	N/A	N/A	N/A	N/A	N/A
Datta et al. 1999	Review	N/A	N/A	N/A	N/A	N/A	N/A
Bar-Joseph et al. 2002	Observational	Low	Low	Low	Low	Low	Low
Bar-Joseph et al. 2005	Observational	Low	Low	Low	Low	Low	Low
Vukmir & Katz 2006	RCT	Low	Low	Low	Low	Low	Low
Spohr et al. 2008	Review	N/A	N/A	N/A	N/A	N/A	N/A
Weng et al. 2013	Observational	Low	Low	Low	Low	Low	Low

In 1990, Roberts and colleagues published a retrospective study in which the researchers attempted to determine mortality predictors during cardiac arrest in resuscitated patients. The results showed that out of the 238 patients studied, only 10 survived, giving a 4.2% survival rate when SB was used in resuscitation [15]. This low survival rate was attributed to severe illness due to severe MA in patients who needed SB during the procedure. Similarly, in an observational cohort study conducted in 1990 on IHCA and OHCA patients who were administered epinephrine as per the ACLS guidelines to determine the correlation between ACLS drugs, including SB [16], and one-hour survival rate to hospital discharge, the researchers established that SB had no significant correlation with survival, but the timing of administration of the drug was a critical factor, i.e., early administration of SB is critical to preventing the development of severe MA [17].

In addition, in 1995, in an RCT by Dybvik et al. involving 502 adults resuscitated after OHCA with failed initial defibrillation attempt, 245 of the patients were given SB mixed with trometamol-phosphate (250 mL) compared to the 257 patients who were given 0.9% saline solution (250 mL) during resuscitation [18]. The trial showed that buffer therapy had no significant improvement in the outcome for patients who experienced MA during resuscitation after OHCA. In another similar study, Walraven in 1998 failed to establish the relationship between ACLS medications, including SB, and survival rates, with the researchers concluding that there was no correlation between SB and successful resuscitation [19]. Nonetheless, Adgey and Johnston in 1999 reviewed the existing literature and concluded that buffer solutions used during resuscitation after cardiac arrest should be limited to incidents of extreme acidosis and ought to be administered only after protracted resuscitation attempts or in cases with hyperkalemic cardiac arrest or overdose due to tricyclic antidepressant [20].

In the same year, Datta et al. conducted a review to evaluate advanced life support, post-resuscitation care, and life support [21]. The authors recommended that SB ought not to be employed routinely in patients with cardiac arrest due to insufficient evidence to support its beneficial use in cardiac arrest patients. In 2022, Bar-Joseph et al. carried out a large retrospective study with 2,915 patients based on clinical trial III brain resuscitation to determine the relationship between SB use and ACLS duration [22]. The researchers found a linear relationship between SB use and ACLS duration and recommended that SB administration should be started early due to the development of severe MA. However, in their second study three years later, contradictory results were obtained for OHCA patients in which the duration from the time of the cardiac arrest to ACLS initiation was found to be less than 30 minutes and that early and more recurrent administration of SB lead to improved ROSC and better long-term neurologic outcomes [23].

Moreover, in the next year, Vukmir and Katz conducted a prospective RCT among 792 patients with pre-hospital cardiac arrest before defibrillation was integrated into clinical practice. The duo established that a difference existed in survival rates between patients who received SB after ACLS protocol (58/420, 13.9%) and those who received normal saline (52/320, 13.8%) [24]. However, patients with prolonged pre-hospital cardiac arrest (>15 minutes) had higher survival rates (32.8%) in the SB group compared to 15.4% in the control group. Similar results were obtained in 2013 by Weng et al. who noticed that patients who were given SB in a protracted resuscitation had a greater percentage of ROSC than those who did not, but there was no significant increase in ROSC rate in OHCA patients [25]. Spohr et al. also conducted a systematic review to determine which ACLS medications were beneficial for short-term survival following a heart attack and recommended that SB ought to be administered during CPR only in cases of lengthy failed resuscitation [26].

Touron et al. carried out two retrospective studies according to the analysis of a French and North American dataset and investigated whether prehospital SB administration was associated with better neurologic outcomes. The results showed that SB in OHCA patients was not associated with neurologic outcomes in a French dataset and was associated with worse neurologic outcomes in a North American dataset. [27]

**Figure 2:**
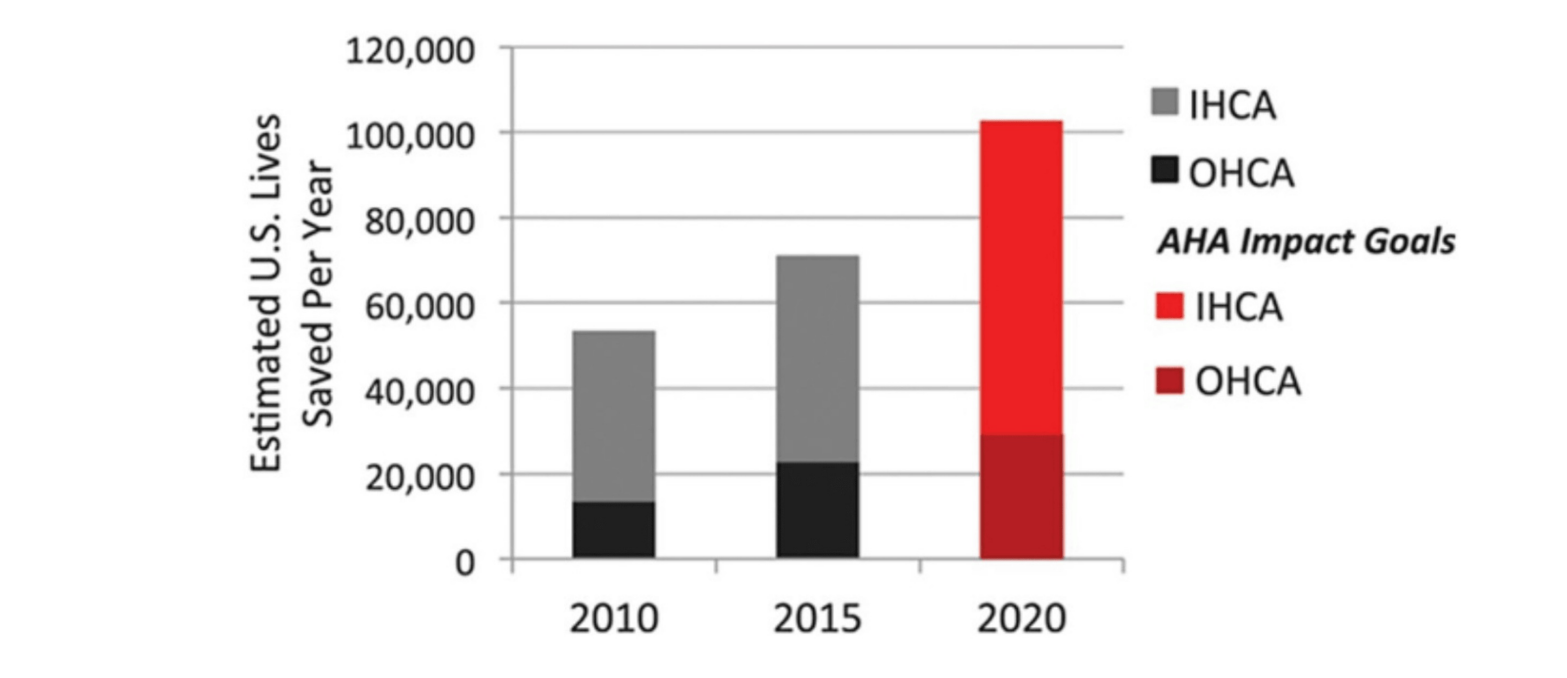
Number of lives saved in the US following sudden cardiac arrest. IHCA, in-hospital cardiac arrest; OHCA, out-of-hospital cardiac arrest; AHA, American Heart Association Source: Adapted from Neumar (2020) [28] Permission has been obtained from the original publishers.

In Figure 2, we can observe the number of lives saved in the US annually following cardiac arrest, which is low considering survival rates to hospital discharge average about 9.6% [28].

When reviewing the clinical records of some 88 patients who were given SB during resuscitation in a retrospective study, Geraci et al. noticed that 31% of the patients (27/88) were given SB without data on arterial blood gas (ABG). For patients whose ABG information was accessible, the administration of SB resulted in alkalemia in 10/61 (16%) of the patients. Therefore, the authors recommended that early analysis of ABG data may reduce the frequency of SB use and help in optimizing pH during cardiac arrest [29]. Moreover, a review by Lee (2011) to determine the role played by the administration of drugs following a successful CPR and defibrillation showed that none of the drugs or their combinations showed beneficial outcomes on long-term survival, while a systematic appraisal reported that short-term results following a successful CPR have become better due to code drugs administration, although no significant benefit was reported in the final outcome [30].

After 2015, only a handful of studies have been conducted on SB during CPR, with most being reviews. One such review was performed by Alshahrani and Aldandan in 2021 using RCTs and observational studies on OHCA adults who were given B during CPR versus those who were not (control) [31]. The authors found no significant difference between patients who received SB and the control group. They also determined that SB was correlated with a lower rate of sustained ROSC compared to the control group, which is contradictory to what Kim et al. and other studies have established since SB and its frequency of use are highly correlated with ROSC [32].

European Resuscitation Council (ERC) guidelines for resuscitation (2021)

The ERC guidelines are specific instructions followed during resuscitation after cardiac arrest and encompass the epidemiology of cardiac arrest, systems that save lives, basic life support (BLS), adult advanced life support (ALS), cardiac arrest in special scenarios, post-resuscitation care, first aid, newborn resuscitation and support of transition of infants at birth, pediatric life support, education for resuscitation, and ethics of resuscitation and end-of-life decisions [33]. In this section, the focus is on ALS, which refers to progressive interventions for cardiac arrest patients after BLS. ALS is usually performed using an automated external defibrillator (AED) and encompasses preventing or treating both IHCA and OHCA, manual defibrillation, the ALS algorithm, treatment of peri-arrest arrhythmias, medications and their administration during CPR, and air management during CPR [33].

For IHCA, the ERC recommends that vasopressor drugs like adrenaline (epinephrine) 1 mg should be given intravenous (IV) or intraosseous (IO) as soon as is plausible to adult patients with a non-shockable rhythm or after the third shock with a shockable rhythm and the procedure should be replicated after every three to five minutes while continuing ALS. Moreover, antiarrhythmic drugs like amiodarone 300 mg should be given IV or IO to adults who are in ventricular fibrillation (VF) or pulses ventricular tachycardia (pVT) after administering the third shock and a further 150 mg IV or IO of amiodarone after the fifth shock [33]. Alternatively, 100 mg IV or IO of lidocaine can be utilized if amiodarone is unavailable and a further 50 mg can be given after the fifth defibrillation attempt. Similarly, when the cause of cardiac arrest is confirmed or suspected to be pulmonary embolus, thrombolytic drugs may be considered and administered followed by a 60-90-minute duration of CPR [33].

Nevertheless, for OHCA, the ERC recommends IV as opposed to IO, due to worse outcomes when the latter route is used but it may be used if access to the former is encountered. Regardless, the vasopressor drug to use is 1 mg adrenaline (vasopressin is not recommended), which significantly improves the survival to hospital admission and the long-term survival of up to three months but has unfavorable neurological outcomes. This can be administered as soon as possible with a non-shockable rhythm or after the third shock with a shockable rhythm and should be replicated every three to five minutes while continuing ALS [33]. In addition, antiarrhythmic drugs to use include amiodarone and lidocaine. Amiodarone 300 mg should be administered after the third defibrillation attempt notwithstanding whether the shocks are consecutive or interrupted when giving CPR. Similarly, lidocaine 100 mg can be utilized if amiodarone is unavailable or when a decision is made to use it instead of amiodarone. This should be followed by a 50 mg bolus after the fifth defibrillation attempt [33]. The use of lidocaine over amiodarone is preferred when ROSC is necessitated for OHCA patients [33,34].

The ERC does not recommend the utilization of thrombolytic drugs for OHCA patients unless pulmonary embolus is conjectured or corroborated as the basis of the condition. If that is the case, the drug should be administered, followed by 60-90 minutes of CPR before halting resuscitation attempts [33]. In addition, under no circumstance does the ERC recommend the utilization of SB during cardiac arrest, owing to its undesirable outcomes and lack of sufficient evidence to support its use.

**Figure 3:**
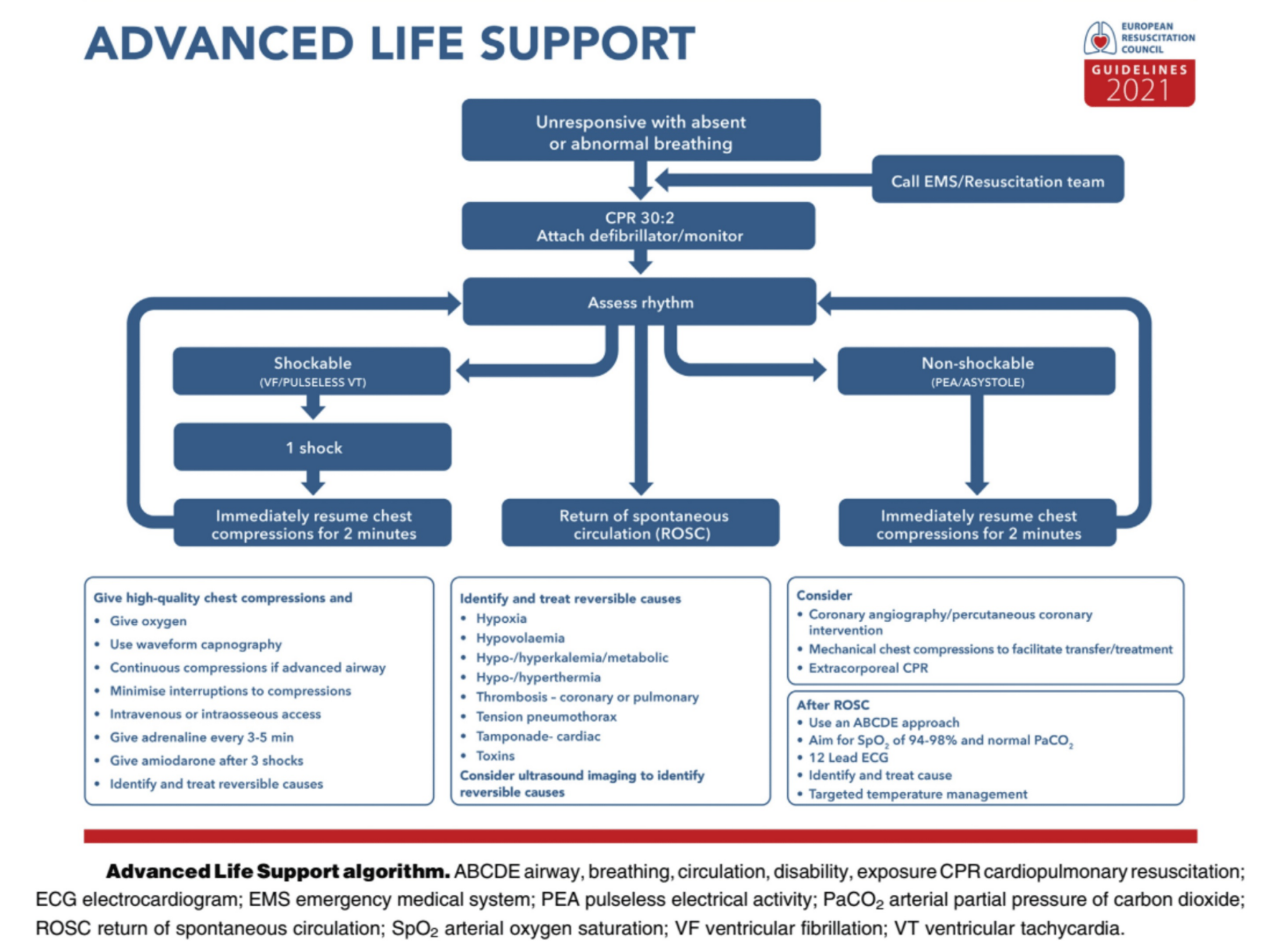
2021 European Resuscitation Council (ERC)’s Advanced Life Support Algorithm. Source: Soar et al. (2021) [35] Permission has been obtained from the original publishers.

Figure 3 summarizes the ALS algorithm as proposed by the 2021 ERC guidelines for resuscitation.

Current guidelines and recommendations by the American Heart Association (AHA) (2020)

The OHCA recommendations regarding the use of SB that have been in existence for about four decades pertain to the limited evidence to back its effectiveness. The medication is believed to combat acidosis by safeguarding against acid-base imbalances that occur during cardiac arrest. A pH < 7.2 is considered severe acidemia and suppresses myocardial contractility, through what many researchers believe to be hyporesponsiveness to vasopressors and inotropes [2]. Hence, SB administration could be suggested as a potentially promising therapy in OHCA to alleviate acidosis, which consequently reduces the risk of injury and death [7].

Recent studies have made recommendations to the AHA to permit but restrict the use of SB to scenarios with cyclic antidepressant overdose, hyperkalemic cardiac arrests, and ventricular arrhythmias [36]. Moreover, the frequency of administration of SB in OHCA has diminished in recent years, yet its prehospital use has remained variable. Regardless, SB has been recommended for use in lengthy intervals of cardiopulmonary resuscitation (CPR, Class IIB), underlying hyperkalemia (Class I intervention), barbiturates overdose (Class IIA), and underlying metabolic acidosis [11,37]. Nevertheless, the 2010 AHA guidelines did not endorse the repetitive use of SB during resuscitation following a cardiac arrest but limited its application to the aforementioned specific scenarios [38].

**Figure 4:**
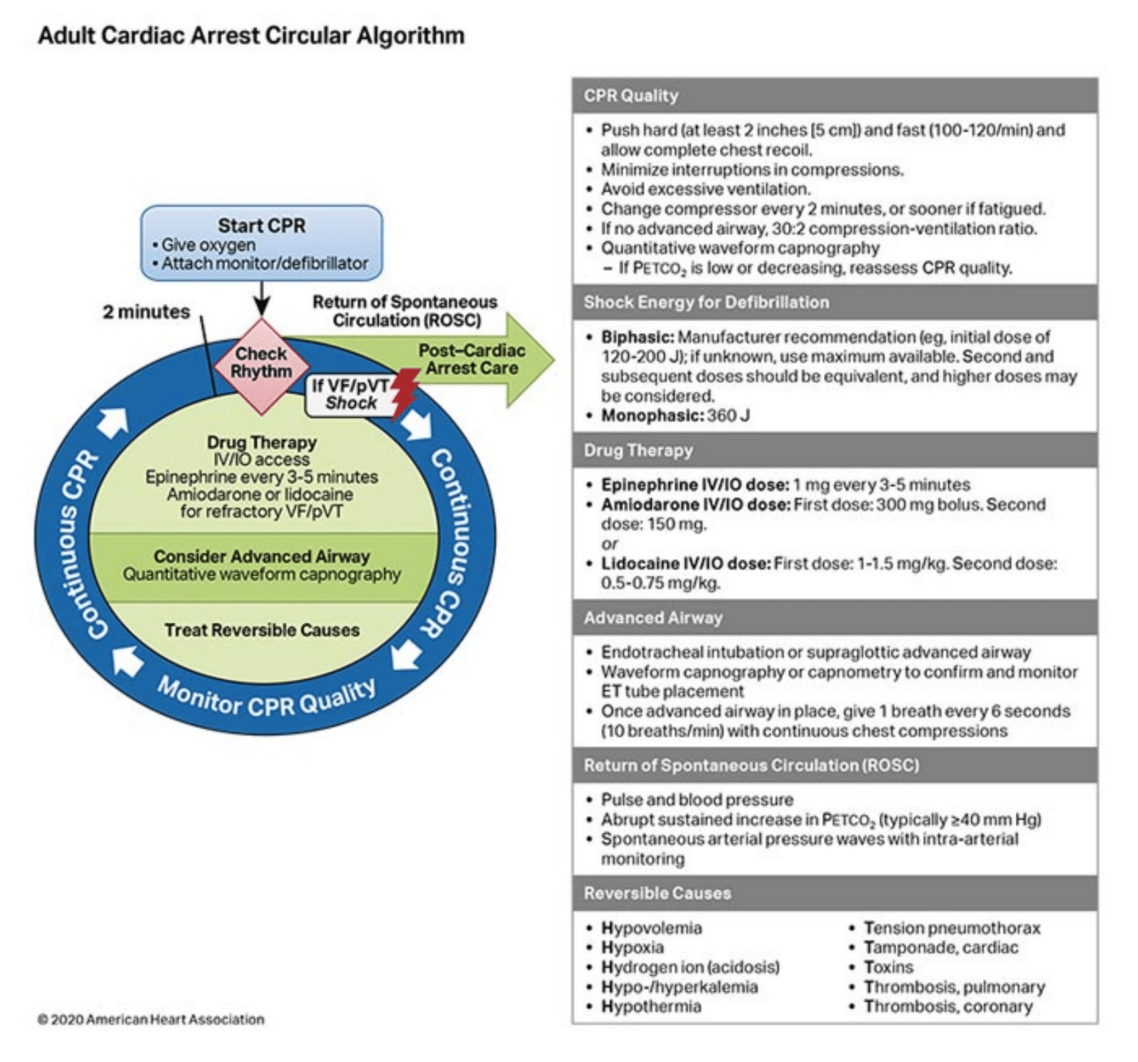
2020 American Heart Association (AHA)’s circular ACLS algorithm. ACLS, Advanced Cardiac Life Support; CPR, cardiopulmonary resuscitation; ET, endotracheal; IO, intraosseous; IV, intravenous; pVT, pulseless ventricular tachycardia; VF, ventricular fibrillation. Source: AHA (2020) [37] Permission has been obtained from the original publishers.

Current AHA guidelines (2020) suggest that the use of SB therapy during resuscitation needs to be directed by the concentration of the SB serum or calculated on the base deficit. However, this recommendation puts cardiac arrest patients treated with SB at more risk of suffering severe MA. Regardless, the AHA stipulates that SB should only be used as a last-ditch medication for patients who are unresponsive to CPR [38]. The AHA’s ACLS algorithm, as shown in Figure 4, encompasses recommendations from pharmacologic treatment, which are graded and grounded on the level of evidence (LOE), the robustness of the recommendation, and the probability of benefit of the recommendation that is critical to facilitating ROSC [8]. Figure 4 summarizes the AHA ACLS algorithm.

Sodium bicarbonate use in IHCA and OHCA

In OHCA, SB has often been used during the resuscitation of patients with prolonged CPR [39]. Prolonged CPR often results in severe acidosis, which may offset hemodynamic instability due to reduced contractility of the left ventricle and compromised sensitivity to catecholamines [11]. The 1976 publication on ACLS guidelines recommended the utilization of SB during resuscitation, but studies conducted from the 1980s to the 2000s have been unsuccessful in providing sufficient evidence of the benefits of the medication during cardiac arrest [37]. However, recent studies have revealed that SB improves the likelihood of ROSC and increases the rates of survival of patients with prolonged cardiac arrest [36].

In addition, in 2021, Gill et al. reported that improved outcomes can be achieved for IHCA patients when epinephrine is administered early for non-shockable rhythms, CPR is initiated early, and defibrillation for shockable rhythms is performed early [9]. Otherwise, mortality is significantly increased when the duration of resuscitation is increased. Therefore, SB is mainly used during cardiac arrest to reduce the possibility of severe metabolic acidosis even though its benefits remain highly controversial [37]. Moreover, the devices used during resuscitation, such as implantable cardioverter defibrillators (ICDs), and AED also play a significant role in establishing ROSC in OHCA patients, enhancing further the outcomes of the use of SB during resuscitation. In addition, some studies about the administration of SB during cardio-cerebral resuscitation found improved ROSC [40].

In a 2018 study conducted by Ahn et al., the authors compared the performance of SB versus normal saline as medications for use during cardiac arrest resuscitation, whose results are shown in Table 3 [11].

**Table 3:** Results and outcomes between NaHCO3 and normal saline use during cardiac arrest. ROSC, return of spontaneous circulation Source: Adapted from Ahn et al. (2018) [11]

	SB (NaHCO_3_)	Normal Saline	p-value
pH at 20 minutes	6.99	6.90	0.04
HCO_3_ at 20 minutes	21.00	8.00	0.01
Sustained ROSC (%)	4.00	16.00	0.35
Survival to hospital admission (%)	4.00	16.00	0.35
Good neurological outcome at one month (%)	0.00	4.00	1.00

After 20 minutes of CPR, arterial blood gas analysis was performed, pH (6.99 vs. 6.90, P = 0.04) and HCO3− (21.00 vs. 8.00 mEq/L, P = 0.01), which revealed higher levels in the study group than in the control group. We also analyzed the sustained ROSC (%) (4.00 vs 16.00, P = 0.35), survival to hospital admission (%) (4.00 vs 16.00, P = 0.35), and good neurological outcome at one month (%) (0.00 vs. 4.00, P = 1.00) with no statistically significant difference between both groups. These results suggest that SB may offset acid-base imbalances without improving resuscitation success or good neurologic survival [11].

The latest research by Myers et al. in 2023 investigated the role of SB in patients with hyperkalemia, cardiac arrest, and significant MA. The researchers concluded that SB should not be used in hyperkalemic patients to lower potassium levels due to limited literature supporting the practice even though it is a common clinical practice during academia. The authors emphasized that SB worsens survival and neurological outcomes and does not increase survival to hospital discharge even when the length of resuscitation or amount of epinephrine is controlled and should not be recommended for cardiac arrest patients regardless of the comorbidities of downtime. Finally, the use of SB in patients with significant MA resulted in a reduced need for the replacement of kidneys in patients with kidney injuries and is not suggested for use in patients with lactic acidemia but recommended for patients with septic academia and acute kidney injury [41].

Scenarios for use or not of sodium bicarbonate in IHCA and OHCA

Despite some studies supporting the use of SB in OHCA, recent studies have failed to reinforce its application in OHCA alluding to the insignificant differences in the outcomes or the poor outcomes and adverse effects associated with its use, which can be explained by reduced resistance to systemic vessels, reduced ability of hemoglobin to release oxygen, hypernatremia, and inactivation of catecholamines that are often administered simultaneously [7,31]. However, in studies where poor outcomes and effects were observed, SB was administered later after the initial resuscitation attempts failed [7]. Therefore, it can be hypothesized that the connection of SB with poor outcomes and adverse effects can be confused with unsuccessful lengthy resuscitation efforts that characteristically have deficient prognoses.

In addition, while some studies have shown that the utilization of SB in OHCA results in improved ROSC and increased rates of hospital admissions, many studies have revealed that its administration is counter-productive due to increased blood carbon dioxide tension in tissues and central veins, resulting in abnormally increased respiratory acidosis [39]. Therefore, the ACLS recommendation after 2010 stated that the routine use of SB for adults should not be applied during CPR, except for hyperkalemia, underlying metabolic acidosis, or overdose with a tricyclic antidepressant [11]. SB has since then been removed as a medication of choice from the ACLS guidelines due to insufficient support from the literature that shows its benefits on the survival rates for patients with cardiac arrest [3].

Moreover, the removal of SB as medication for use during cardiac arrest resuscitation was reinforced by the undesirable and harmful outcomes when SB is used during resuscitation following cardiac arrests, such as the expansion of the extracellular fluid volume, hypernatremia, reduced ionized calcium, metabolic acidosis, reduced vascular resistance, and hyperosmolarity [37].

Most studies involving the efficacy of SB as medication used during CPR have been based on clinical trials involving OHCA patients. In addition, most studies on IHCA patients are heterogeneous, involving patients in critical and non-critical care settings, making the process of care and the cause of and survival from cardiac arrest different for IHCA and OHCA patients [8]. Therefore, the same treatment procedures cannot be applied to the two different groups of patients even with the same medication, such as SB.

Limitations

This review faces several limitations. The included studies exhibit significant heterogeneity in methodologies, populations, and clinical contexts, complicating direct comparisons and synthesis. Study quality varied, with some observational studies showing a high risk of bias, which could impact the reliability of the findings. Many studies needed more detailed information on specific interventions and patient conditions, limiting the depth of analysis and adjustment for confounding factors. In addition, some studies utilized outdated guidelines, which may limit the applicability of their findings to contemporary practice. Despite these limitations, this review highlights critical gaps in the literature. It underscores the urgent need for more rigorous, controlled studies to determine the definitive role of sodium bicarbonate in cardiac resuscitation.

## Conclusions

In cardiac arrest patients, the use of SB was not associated with improvement in survival and neurologic outcomes. Despite this, some studies suggest SB may be administered under specific conditions like hyperkalemic cardiac arrest, severe cardiotoxicity, or tricyclic antidepressant overdose. 

This review concludes there is insufficient evidence to support the routine use of SB in cardiac arrest resuscitation and further studies should be performed to determine its role in these scenarios.

## References

[1] Velissaris D, Karamouzos V, Pierrakos C, Koniari I, Apostolopoulou C, Karanikolas M (2016). Use of sodium bicarbonate in cardiac arrest: current guidelines and literature review. J Clin Med Res.

[2] Jung B, Martinez M, Claessens YE (2019). Diagnosis and management of metabolic acidosis: guidelines from a French expert panel. Ann Intensive Care.

[3] Adeva-Andany MM, Fernández-Fernández C, Mouriño-Bayolo D, Castro-Quintela E, Domínguez-Montero A (2014). Sodium bicarbonate therapy in patients with metabolic acidosis. ScientificWorldJournal.

[4] Garella S, Dana CL, Chazan JA (1973). Severity of metabolic acidosis as a determinant of bicarbonate requirements. N Engl J Med.

[5] Arieff AI, Leach W, Park R, Lazarowitz VC (1982). Systemic effects of NaHCO3 in experimental lactic acidosis in dogs. Am J Physiol.

[6] Benjamin E, Oropello JM, Abalos AM (1994). Effects of acid-base correction on hemodynamics, oxygen dynamics, and resuscitability in severe canine hemorrhagic shock. Crit Care Med.

[7] Niederberger SM, Crowe RP, Salcido DD, Menegazzi JJ (2023). Sodium bicarbonate administration is associated with improved survival in asystolic and PEA out-of-hospital cardiac arrest. Resuscitation.

[8] Benz P, Chong S, Woo S (2020). Frequency of advanced cardiac life support medication use and association with survival during in-hospital cardiac arrest. Clin Ther.

[9] Gill HS, Lindgren E, Steele AD (2021). Errors of commission in cardiac arrest care in the intensive care unit. J Intensive Care Med.

[10] Gräsner JT, Herlitz J, Tjelmeland IB (2021). European Resuscitation Council Guidelines 2021: epidemiology of cardiac arrest in Europe. Resuscitation.

[11] Ahn S, Kim YJ, Sohn CH, Seo DW, Lim KS, Donnino MW, Kim WY (2018). Sodium bicarbonate on severe metabolic acidosis during prolonged cardiopulmonary resuscitation: a double-blind, randomized, placebo-controlled pilot study. J Thorac Dis.

[12] Wang CH, Huang CH, Chang WT (2016). The effects of calcium and sodium bicarbonate on severe hyperkalaemia during cardiopulmonary resuscitation: a retrospective cohort study of adult in-hospital cardiac arrest. Resuscitation.

[13] Alfonzo A (2016). Survival after in-hospital hyperkalaemic cardiac arrest--does intravenous calcium or sodium bicarbonate influence outcome?. Resuscitation.

[14] Moher D, Liberati A, Tetzlaff J, Altman DG (2009). Preferred reporting items for systematic reviews and meta-analyses: the PRISMA statement. PLoS Med.

[15] Haddaway NR, Page MJ, Pritchard CC, McGuinness LA (2022). PRISMA2020: An R package and Shiny app for producing PRISMA 2020-compliant flow diagrams, with interactivity for optimised digital transparency and Open Synthesis. Campbell Syst Rev.

[16] Roberts D, Landolfo K, Light RB, Dobson K (1990). Early predictors of mortality for hospitalized patients suffering cardiopulmonary arrest. Chest.

[17] Stiell IG, Wells GA, Hebert PC, Laupacis A, Weitzman BN (1995). Association of drug therapy with survival in cardiac arrest: limited role of advanced cardiac life support drugs. Acad Emerg Med.

[18] Dybvik T, Strand T, Steen PA (1995). Buffer therapy during out-of-hospital cardiopulmonary resuscitation. Resuscitation.

[19] Walraven C, Stiell IG, Wells GA, Hébert PC, Vandemheen K (1998). Do advanced cardiac life support drugs increase resuscitation rates from in-hospital cardiac arrest? The OTAC Study Group. Ann Emerg Med.

[20] Adgey AA, Johnston PW (1998). Approaches to modern management of cardiac arrest. Heart.

[21] Datta S, Nasr NF, Khorasani A, Datta R (1999). Current concepts in cardiopulmonary resuscitation in adults. J Indian Med Assoc.

[22] Bar-Joseph G, Abramson NS, Jansen-McWilliams L (2002). Clinical use of sodium bicarbonate during cardiopulmonary resuscitation--is it used sensibly?. Resuscitation.

[23] Bar-Joseph G, Abramson NS, Kelsey SF, Mashiach T, Craig MT, Safar P (2005). Improved resuscitation outcome in emergency medical systems with increased usage of sodium bicarbonate during cardiopulmonary resuscitation. Acta Anaesthesiol Scand.

[24] Vukmir RB, Katz L (2006). Sodium bicarbonate improves outcome in prolonged prehospital cardiac arrest. Am J Emerg Med.

[25] Weng YM, Wu SH, Li WC, Kuo CW, Chen SY, Chen JC (2013). The effects of sodium bicarbonate during prolonged cardiopulmonary resuscitation. Am J Emerg Med.

[26] Spöhr F, Wenzel V, Böttiger BW (2008). Thrombolysis and other drugs during cardiopulmonary resuscitation. Curr Opin Crit Care.

[27] Touron M, Javaudin F, Lebastard Q (2022). Effect of sodium bicarbonate on functional outcome in patients with out-of-hospital cardiac arrest: a post-hoc analysis of a French and North-American dataset. Eur J Emerg Med.

[28] Neumar RW (2016). Doubling cardiac arrest survival by 2020: achieving the American Heart Association Impact Goal. Circulation.

[29] Geraci MJ, Klipa D, Heckman MG, Persoff J (2009). Prevalence of sodium bicarbonate-induced alkalemia in cardiopulmonary arrest patients. Ann Pharmacother.

[30] Lee SW (2011). Drugs in resuscitation: an update. Singapore Med J.

[31] Alshahrani MS, Aldandan HW (2021). Use of sodium bicarbonate in out-of-hospital cardiac arrest: a systematic review and meta-analysis. Int J Emerg Med.

[32] Kim J, Kim K, Park J (2016). Sodium bicarbonate administration during ongoing resuscitation is associated with increased return of spontaneous circulation. Am J Emerg Med.

[33] Lott C, Truhlář A, Alfonzo A (2021). European Resuscitation Council Guidelines 2021: cardiac arrest in special circumstances. Resuscitation.

[34] Kudenchuk PJ, Newell C, White L, Fahrenbruch C, Rea T, Eisenberg M (2013). Prophylactic lidocaine for post resuscitation care of patients with out-of-hospital ventricular fibrillation cardiac arrest. Resuscitation.

[35] Soar J, Böttiger BW, Carli P (2021). European Resuscitation Council Guidelines 2021: adult advanced life support. Resuscitation.

[36] Chen YC, Hung MS, Liu CY, Hsiao CT, Yang YH (2018). The association of emergency department administration of sodium bicarbonate after out of hospital cardiac arrest with outcomes. Am J Emerg Med.

[37] Wu KH, Chang CY, Chen YC, Chang CP, Hsiao CT, Weng HH (2020). Effectiveness of sodium bicarbonate administration on mortality in cardiac arrest patients: a systematic review and meta-analysis. J Emerg Med.

[38] Panchal AR, Bartos JA, Cabañas JG (2020). Part 3: adult basic and advanced life support: 2020 American Heart Association guidelines for cardiopulmonary resuscitation and emergency cardiovascular care. Circulation.

[39] Kawano T, Grunau B, Scheuermeyer FX (2017). Prehospital sodium bicarbonate use could worsen long term survival with favorable neurological recovery among patients with out-of-hospital cardiac arrest. Resuscitation.

[40] Celik T, Ozturk C, Balta S, Erdogan S, Rreka A, Iyisoy A (2016). Sodium bicarbonate dilemma in patients with out-of-hospital cardiac arrest: A double-edged sword. Am J Emerg Med.

[41] Myers V, Mastoras G, Lin S, Gray S (2023). Just the facts: sodium bicarbonate usage in the emergency department. CJEM.

